# Maternal and Perinatal Outcomes of Singleton Term Breech Vaginal Delivery at a Tertiary Care Center in Nepal: A Retrospective Analysis

**DOI:** 10.1155/2020/4039140

**Published:** 2020-11-16

**Authors:** Tulasa Basnet, Baburam Dixit Thapa, Dipti Das, Ramesh Shrestha, Sarita Sitaula, Anu Thapa

**Affiliations:** ^1^Department of Obstetrics and Gynecology, B. P Koirala Institute of Health Sciences (BPKIHS), Dharan, Nepal; ^2^B and B Medical Institute, Lalitpur, Nepal

## Abstract

**Background:**

Breech presentation is associated with increased rates of maternal and perinatal morbidity regardless of mode of delivery. After the results of Term Breech Trial, most of the countries adopted the protocol of cesarean section for term breech delivery because of which breech vaginal delivery is becoming rare. The aim of this study is to evaluate short-term maternal and perinatal outcomes of breech vaginal delivery at a tertiary care hospital in Nepal.

**Methods:**

A retrospective review of case records of all women who had vaginal breech delivery from April 13, 2016, to April 12, 2018, was conducted, over a period of two years. Available demographic variables, obstetric characteristics, details of labor, postpartum complications, and perinatal complications were recorded and analyzed.

**Results:**

Out of 21,768 cases of deliveries during the study period, the incidence of term breech deliveries was 528 (2.4%) among which the mode of only 84 (17.8%) deliveries was vaginal. Most of the deliveries were unplanned and were conducted because emergency cesarean section could not be performed. Three (3.6%) women had postpartum hemorrhage, and four (4.8%) had entrapment of aftercoming head, two of them requiring Dührssen incisions. Adverse perinatal outcomes were seen in 23.8% of such deliveries with <7 APGAR score at 5 minutes in 20.2%, neonatal admission in 17.7%, and perinatal mortality in 8.3%. The perinatal mortality was significantly associated with birthweight less than 2500 grams as compared to birthweight ≥2500 grams (21.1% versus 4.6%; *P*=0.043).

**Conclusion:**

The perinatal outcomes for vaginal breech delivery are grave with our existing health facilities, especially when the deliveries are not well planned.

## 1. Introduction

The incidence of breech presentation at term among singleton pregnancies is 3–5% [1]. Increased rates of maternal and perinatal morbidity are associated with breech presentation regardless of mode of delivery [2]. Complications like genital tract injuries are more common with breech presentation in both vaginal and cesarean delivery in the case of mother, while, for fetus, the common risk associated with breech presentation is premature delivery, umbilical cord prolapse, and birth trauma.

There has always been a controversy over the optimal mode of delivery regarding singleton term breech presentation [3]. Vaginal breech delivery is associated with a 10-fold higher risk of intrapartum fetal death as compared to vaginal cephalic delivery [4]. Overall, the risk of perinatal mortality for planned vaginal breech delivery is approximately 2/1000. Similarly, the risk is 1/1000 for cephalic vaginal delivery and 0.5/1000 for cesarean section after 39 weeks [5]. These rates, however, can vary according to healthcare practices and available facilities, socioeconomic condition, and many other factors related to health care delivery systems.

The results of “Term Breech Trial (TBT)” showed that the planned cesarean delivery for singleton term breech presentation is associated with lower perinatal mortality and serious perinatal morbidity in comparison to vaginal delivery [6]. Owing to this contextual benchmark, the subsequent practices including contemporary ones have sidelined with the findings of the trial resulting in a consistent endorsement of elective cesarean section policy for term breech delivery by most of the health facilities. The follow-up studies of TBT, however, for both maternal and fetal outcomes showed similar results in both cesarean and vaginal delivery groups thereby concluding that planned cesarean delivery does not reduce the risk of death or neurodevelopmental delay in children [7, 8]. Furthermore, perinatal or neonatal mortality and severe neonatal morbidity were not reduced even with the policy of elective cesarean section for term breech delivery in settings with high national perinatal mortality rate [9].

In low-income countries like Nepal, cesarean section for all breech deliveries may not be feasible because of limited surgical infrastructure in most health facilities as well as high cost implication borne by patients themselves and without any third party medical coverage. Regarding this, the American College of Obstetricians and Gynecologists (2016b) as well as Royal College of Obstetricians and Gynecologists (RCOG, 2017) recommend that the risks and benefits of both modes of delivery should be discussed with the patient. They also suggest that an external cephalic version should be offered to women with breech presentation at term if there are no contraindications [5, 10]. Moreover, it is explicated that the decision regarding the mode of delivery should be contingent upon the expertise of the healthcare providers and also that planned term breech vaginal delivery may be reasonable under hospital-specific protocol [10]. This study aimed to find out the short-term maternal and perinatal outcomes of term breech vaginal delivery.

## 2. Methods

### 2.1. Study Setting

This study was carried out at B. P. Koirala Institute of Health Sciences (BPKIHS), which is a tertiary care hospital situated in the eastern region of Nepal. The hospital provides obstetrics and gynecologic services to the women of province number one (heretofore unnamed) as well as some parts of India. Out of 10,000 to 12,000 deliveries that are conducted in the hospital annually, only around 40% of women seeking delivery are registered or booked to the hospital. The rest are unregistered, few are referred, and many present without even a single antenatal visit. The specific protocol guideline of the institute is to opt for a cesarean section for term breech delivery. Despite that, a significant number of vaginal breech deliveries are also conducted because most of these patients are unregistered due to which elective cesarean section cannot be planned on time; many arrive in advanced stages of labor; and some refuse cesarean delivery owing to financial and social issues.

Being an academic institute with comprehensive indulgence in medical education in both undergraduate and postgraduate levels, monitoring and further supervision for case-specific delivery complications are handled and sought after accordingly. Vaginal deliveries are usually conducted by OBGYN resident doctors and nursing staffs posted in labor room. For high risk cases like breech vaginal deliveries, resident doctors conduct the deliveries supervised by lecturers/senior residents. In cases of more difficult presentations, immediate assistance from consultant on call is sought.

### 2.2. Study Design

**Figure 1:**
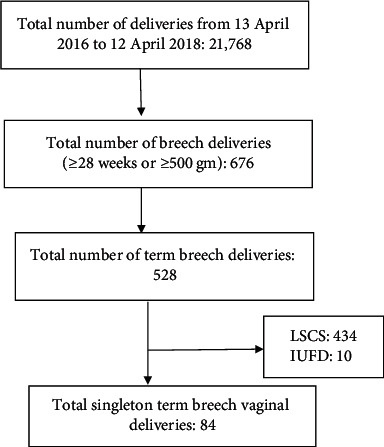
Selection of study population.

The study was carried out through a retrospective analysis and review of two years of case records of women who had singleton term breech vaginal delivery from April 13, 2016, to April 12, 2018. The women with live singleton pregnancy with breech presentation between 37 and 42 weeks of gestation were included in the study. The study excluded the women with preterm deliveries (<37 weeks), postterm pregnancies (>42 weeks), prediagnosed IUFD, and multifetal pregnancies. Women with postterm pregnancies in particular were excluded from the study because postterm births themselves are associated with increased perinatal morbidities. However, in our cohort, there were no women with breech presentation who delivered after 42 weeks. The process of selection of the study population is shown in Figure 1. The available information was recorded in preformed pro forma. Ethical approval was taken from the Institutional Review Committee (IRC Code: IRC/1477/018) before conducting the study and permission was obtained from the hospital director to review the case records. The case variables studied were as follows:

  Demographic variable: age
  Obstetric characteristics: antenatal care (registered or unregistered), parity, gestational age, antenatal complications (GDM, hypertensive disorder, anemia, IUGR, and oligohydramnios)
  Details of labor: total duration of labor, duration of second stage of labor, prelabor rupture of membrane, umbilical cord prolapse, need of episiotomy, perineal tear, other genital tract injuries, and birthweight
  Postpartum complications: postpartum hemorrhage and maternal mortality
  Perinatal complications: APGAR at 5 minutes <7, neonatal admission, and perinatal mortality

### 2.3. Data Analysis

The data was entered in the master chart and analysis was performed using SPSS 11.5. Descriptive statistics like frequency, percentages, and mean and standard deviation were calculated and the results were presented in tables. Association between the maternal and fetal characteristics and perinatal outcomes was estimated using Chi-square test and Fisher exact test where applicable. *P* < 0.05 was considered statistically significant.

### 2.4. Definition of the Key Terms

Term pregnancy: includes early term (37°^/7^ weeks to 38^6/7^ weeks), full term (39°^/7^ weeks to 40^6/7^ weeks), and late term (41°^/7^ weeks to 41^6/7^ weeks) gestation.

  Still birth: when signs of life are absent at birth. It includes both intrauterine and intrapartum death. Only intrapartum still birth taken into consideration for the study.
  Early neonatal death: death of live born neonate during the first seven days of life.
  Perinatal death: intrapartum still births plus early neonatal deaths.
  Adverse perinatal outcomes: included low APGAR score at 5 minutes (<7), neonatal admission, and perinatal death.

## 3. Results

There were 21,768 deliveries over the study period of two years. Total 676 breech deliveries occurred over this period, with an incidence of 3.1% of the total deliveries. Among them, 528 were term breech deliveries (between 37 and 42 weeks of gestation), accounting for 2.4% of the total deliveries. Out of 528 term breech deliveries, 434 (82.2%) women delivered through cesarean section and 94 (17.8%) of them vaginally. Ten women were excluded as they had been diagnosed with IUFD at presentation. Therefore, 84 women meeting the inclusion criteria were taken for the analysis.

For 66 (78.6%) women, vaginal breech delivery was unplanned. Thirty-six (42.9%) women presented in second stage and seven women were already in advanced stage of labor. For the remaining 23 women, even though the LSCS was planned, it could not be performed immediately, mostly because of preoccupied operation theatre. Some women, however, opted for vaginal delivery; among the 18 (21.6%) women, two had contraindications for vaginal delivery (one had oligohydramnios with IUGR; the other had IUGR) and were counseled against going for vaginal delivery. But those women did not give consent for cesarean section. They eventually had vaginal delivery with poor perinatal outcomes.

The mean age of the women in study was 25.67 ± 5.06 years and most of the women were within the age groups 20–30 years. The mean gestational age at delivery was 39 weeks with 15 (17.9%) women crossing the expected date of delivery; none of the women were beyond 42 weeks of gestation. Antenatal complications were present in 14 (16.7%) women. Among them, the most common was hypertensive disorder of pregnancy, present in seven women; four women had GDM; three had oligohydramnios; two had IUGR; and one each had anemia and antepartum hemorrhage. The demographic and obstetric characteristics of the women are presented in Table 1.

As more than two-thirds of the women visiting the center presented in the advanced stage of labor, exact duration of the labor could not be determined. For a rough estimation, however, the tentative period of the commencement of labor pain to delivery was noted. The details of labor as well as maternal complications are presented in Table 2. There were no cases of instrumental deliveries or III- and IV-degree perineal tear. Having said that, 63.1% women required episiotomy and two women required Dührssen incision for the delivery of entrapped aftercoming head. The details of the women experiencing entrapment of aftercoming head are presented in Table 3. There were no maternal mortalities. Among three women experiencing PPH, the etiology of two was atonic and one was traumatic.

The perinatal outcomes are summarized in Table 4. Adverse perinatal outcomes (low APGAR at 5 minutes, neonatal admission, and perinatal mortality) were reported in 23.8% of deliveries. Fourteen (17.7%) neonates required admission in different wards, three with diagnosis of birth asphyxia (5 min APGAR ≤3), and others for respiratory distress. There were seven cases of perinatal mortality (five still births and two early NND) giving a mortality rate of 8.3% of the total term vaginal breech deliveries.

The association was sought between the independent variables: maternal age, parity, booking status, period of gestation, birthweight, and perinatal outcomes against APGAR at 5 min, neonatal admission, and perinatal mortality. The results are presented in Table 5. We also tried to find out the association between labor duration and perinatal outcomes applying logistic regression. A negative association was observed between duration of second stage of labor and perinatal outcomes (regression coefficient −0.041, *P*=0.178).

## 4. Discussion

This retrospective study is aimed at determining the short-term maternal and perinatal outcomes of singleton breech vaginal delivery at a tertiary level hospital in a low resource country. The study reported very high rate of adverse perinatal outcomes (23.8%) as well as perinatal mortality (8.3%).

Large population based studies are not available to quote the incidence of breech deliveries in our setup. Hospital-based studies conducted in different tertiary level hospitals have reported the breech delivery incidence of 1.9–2.5% [11, 12], similar to the incidence reported in our study. But this is slightly lower than the overall incidence of 3–5% [1]. The proportion of women undergoing vaginal breech delivery is minuscule because the institute has adopted the protocol of performing cesarean section for all term breech deliveries. For women who seek vaginal delivery, counseling is done regarding the hospital protocol, known contraindications, and risks associated with it. If woman gives consent after thorough briefing of all the possible complications, vaginal delivery is conducted. But almost all women opt for cesarean section after counseling. Also, because of unavailability of adequate human resources and tools for intrapartum fetal monitoring as well as lack of skills for vaginal breech deliveries, even healthcare providers prefer performing cesarean section for breech deliveries. As predicated by the study, most of the vaginal deliveries occurred because they were imminent or the operation theatre was unavailable at the moment. Nearly 80% of women are unregistered and the appropriate mode of delivery could not be planned for them beforehand. This led to many unplanned vaginal breech deliveries which in part is responsible for the high rate of adverse perinatal outcome. Nevertheless, the prevalence is not homogeneous all over the country; hospitals have reported up to 40–52% of vaginal deliveries among breech presentation with perinatal outcomes similar to or even superior to cesarean deliveries [12, 13].

In TBT, adverse maternal outcomes which included maternal mortality or serious morbidity were noted in 3.2% women among planned vaginal delivery group. Postpartum hemorrhage was reported in 1.3% women [6]. The adverse maternal outcome we observed was postpartum hemorrhage which presented in 3.6% women. Out of three women who had PPH, two had atonic PPH and one had traumatic PPH secondary to the application of cervical incision to deliver entrapped head. Similar incidence of PPH was reported by Wasim and colleagues [14] but higher rate (13.2%) is reported in the study by Dohbit et al. [15].

Entrapment of aftercoming head is a specific intrapartum emergency associated with breech vaginal delivery and it reflects either incompletely dilated cervix or cephalopelvic disproportion [1]. This complication is more common in preterm breech deliveries and can occur in both vaginal and cesarean deliveries. Kayem et al. reported head entrapment in 13.1% among vaginal and 5.9% among cesarean deliveries of preterm breech [16]. Out of four women with this complication, two had contraindications for vaginal breech delivery; one had oligohydramnios; and for both of them IUGR was missed at the time of admission. The duo were planned for cesarean section; however, it could not be performed timely. The other two women presented in second stage of labor, one already with entrapped head. The perinatal outcome for all four deliveries was poor. All these deliveries were being monitored closely in the presence of senior residents. In two of the cases, MSV maneuver was effective in delivering head, while, for the remaining two, head could not be delivered with the maneuver alone, so help from duty consultant was sought resulting in successful delivery after the application of Dührssen's incision in the cervix.

Term babies with breech presentation are reported to have worse outcomes than cephalic ones, irrespective of the mode of delivery [2]. In our study, adverse perinatal outcomes were reported in nearly one fourth of deliveries. Azria et al. and PREMODA Study Group reported adverse perinatal outcome in 6.59% cases [17]. The criteria for adverse perinatal outcomes used were similar but we included only three variables in contrast to eight variables used by the study group. The lesser rate of morbidity in their study can be attributed to the preregistration and planning of the cases in advance and also the inclusion of only certain interventions as adverse outcomes in contrast to our methodology which has also taken into account the prospects of hospital admission as an attributable variable. Perinatal mortality or serious neonatal morbidity was reported in 5.0% of cases in vaginal delivery group in TBT [6]. Higher rate of adverse events was noted in women with age >30 years, gestation more than 40 weeks, and birthweight less than 2500 grams. Increased duration of second stage was also negatively associated with perinatal outcomes.

Our analysis reported a very high perinatal mortality among vaginal breech deliveries compared to other studies [11, 12, 15]. Mortalities were higher among those with gestation more than 40 weeks, or among babies weighing less than 2500 grams as compared to those with birthweight ≥2500 grams (21.05% versus 4.62%; *P*=0.043). Conde-Agudelo et al. on analyzing the fetal deaths also reported that risk of fetal death is minimum at 39 weeks which gradually increases with the period of gestation [4]. Some of the guidelines also recommend vaginal breech delivery only if the estimated fetal weight is between 2500 and 4000 grams to avoid growth restricted fetuses to undergo vaginal delivery [18].

Out of seven mortalities, three women who presented in LSOL and the rest in second stage of labor; none of them had prolonged labor. However, all these mortalities may not be solely associated with the mode of delivery. In two women, no other complications were identified which could have increased the chances of mortality. Two women had entrapment of aftercoming head in second stage of labor, one of which was presented to the facility with an already entrapped head in the second stage. Three women had associated obstetric complications: one had oligohydramnios with IUGR (weight 1530 grams); another had anemia and hypertensive disorder with IUGR (weight 1550 grams); and the third one had GDM. These conditions were identified at the time of delivery. Although cesarean section was planned for the first two women, failure to achieve consent resulted in vaginal breech delivery. Cases like this have been found to affect the obstetric outcomes. The treatment cost is to be borne by the patients themselves. The unmatched cost of cesarean section, hospital stay, and required medications vis-à-vis the average Nepalese income is major cause of women refusing cesarean section. Furthermore, the babies delivered from such mothers require NICU which itself is an infrastructural challenge because of limited institutes with neonatal intensive care units, the logistics associated to referral, and also the spatial difficulties with such cases. This further adds to the financial burden hitherto experienced by the patients and parties demotivating the aspiration of adopting and implementing an optimal protocol in the delivery procedure. The socioeconomic reliability of Nepalese people is generally on agriculture and animal husbandry which are highly unpredictable occupations requiring number of hours of labor time. The incidence of prolonged hospital stay as well as the need for recuperative respite for a longer duration is significantly higher in case of surgical intervention as compared to vaginal delivery which in turn delays their return to work. This deems them twice-disadvantaged: one from the hospital and recuperative cost and the other from employment struggles. Because of these financial and social issues, many women and their family members are reluctant to opt for cesarean section and prefer vaginal delivery at any cost. Though we cannot derive a conclusion solely based on this observation, these financial and social issues nevertheless play a major role in poorer maternal and perinatal outcomes as witnessed in our cases.

The higher rates of perinatal morbidity and mortality associated with vaginal breech deliveries in our study are not in compliance with results from other hospitals of Nepal [11–13]. This may be because of insufficient sample size in study—that those hospitals have lower numbers of total deliveries which allows them to allocate adequate human resources for the management of those cases. But well planned vaginal deliveries could also be a justification in addition to well-managed skilled resources resulting into high rate of breech vaginal deliveries in those institutions.

Following the results of this study, demonstration classes on vaginal breech deliveries have been conducted for the residents. Proper screening of the women at presentation for feasibility of vaginal delivery is assured by senior resident or consultant on duty. Also, the hospital management is requested to provide another operating room on need basis so that the women who are not suitable will not have to go for vaginal delivery because of unavailability of operating room. With these measures, we hope to experience better outcomes of vaginal breech deliveries in future.

### 4.1. Limitation of the Study

The current study is a retrospective analysis and has a small sample size. The population studied was heterogeneous and most of the deliveries occurred because they were inevitable rather than planned. Another limitation of the study is that we did not compare the outcomes of vaginal delivery with cesarean delivery. Because of this, we cannot make the inference from the study that the higher rate of perinatal morbidity and mortality can be attributable to vaginal mode of delivery. The study notwithstanding gave us an idea about how our current healthcare delivery for breech presentation at term is working and thus highlighted the scope for improvement.

## 5. Conclusion

The perinatal outcomes for vaginal breech delivery are grave with our existing health facilities and contemporary practices especially when the deliveries are not well planned. Vaginal breech delivery demands special skills, but with decreasing proportion of it, skill transfer to healthcare providers is also plummeting. This is markedly more important for low-income countries like ours where the facility for cesarean section is limited and tertiary care hospitals get overcrowded because of which patients may not get pertinent attention and intervention on time. Conclusively, larger and comprehensive studies comparing outcomes of vaginal and cesarean deliveries should follow in order to reach an incontrovertible and definitive conclusion.

## Figures and Tables

**Table 1:** Demographic and obstetric characteristics.

Characteristics	Frequency	Percentage	Mean ± Std. deviation
*Age (years)*			
<20 years	8	9.5	25.67 ± 5.06
20–30 years	64	76.2	
>30 years	12	14.3	

*Parity*			
Nullipara (parity 0)	39	46.4	
Primipara (parity 1)	32	38.1	
Multipara (parity >1)	13	15.5	

*Antenatal care*			
Registered	19	22.6	
Unregistered	65	77.4	

*Period of gestation at the time of delivery*			
37–40 weeks	69	82.1	39 weeks ±9.4 days
40^1/7^–42 weeks	15	17.9	

*Birthweight (gm)*			
<2500 gm	19	22.6	2753.95 ± 491.02 (1530–3900 gm)
2500–3500 gm	58	69.1	
>3500 gm	7	8.3	
Presence of antenatal complications	14	16.7	

**Table 2:** Details of labor and maternal complications.

Labor details and maternal complications	*N* (%)/Median (25^th^, 75^th^ percentile)
Undiagnosed breech presentation at presentation	1 (1.1)
*Stage of labor at presentation*	
Not in labor	7 (8.3)
Latent stage of labor	21 (25.0)
Active stage of labor	20 (23.8)
Second stage of labor	36 (42.9)
Prelabor rupture of membrane (PROM)	25 (29.8)
Need of episiotomy	53 (63.1)
Cord prolapse	4 (4.8)
Entrapment of aftercoming head	4 (4.8)
Need of cervical incision	2 (2.4)
Postpartum hemorrhage	3 (3.6)
Total labor duration (hours)	7.6 (5.6, 10.6)
Duration of second stage of labor (minutes)	10.5 (7, 18)

**Table 3:** Details of women having entrapment of aftercoming fetal head.

Details	Patient 1	Patient 2	Patient 3	Patient 4
Parity	Nullipara	Nullipara	Nullipara	Nullipara
Registered/Unergistered	Unregistered	Unregistered	Unregistered	Unregistered
Period of gestation	40 weeks	41^2/7^ weeks	40 weeks	40^6/7^ weeks
Presentation and events	Not in labor at presentation. Admitted to the ward and planned LSCS the next day. Went into spontaneous labor and had precipitate labor (total duration 1 hour 30 minutes)	Presented in second stage of labor with entrapped head, had expulsion of trunk 15 minutes prior to presentation to hospital, cord pulsation present during admission	Presented in second stage of labor	Presented in latent stage of labor, progressed while waiting for LSCS due to busy OT
Other antenatal complications	Oligohydramnios. IUGR (missed at the time of admission).	None	Hypertensive disorder	IUGR (missed at the time of admission)
Duration of second stage	18 minutes	Not known	Not known	54 minutes
Delivery of head	By applying maneuvers	Maneuvers failed and delivered by giving Dührssen incision at cervix	By applying maneuvers	Maneuvers failed and delivered by giving Dührssen incision at cervix
*Perinatal outcome*				
Alive/still birth	Alive	Still birth	Alive	Still birth
Birthweight	2000 grams	3750 grams	3350 grams	2100 grams
APGAR at 5 minutes	4	0	4	0
Admission	In NICU		Referred to other health facility for unavailability of NICU bed at the center	
Postpartum complication	None	Postpartum hemorrhage (traumatic)	None	None

**Table 4:** Perinatal outcomes of vaginal breech delivery.

Perinatal outcomes	Frequency	Percentage (%)
APGAR at 5 minutes < 7	17	20.2
Neonatal admission (*n* = 79)	14	17.7
Perinatal mortality (intrapartum still birth and early NND)	7	8.3

**Table 5:** Association of maternal fetal characteristics with perinatal outcomes.

Perinatal outcome										
Maternal and fetal characteristics		APGAR score at 5 minutes		*P* value	Neonatal admission (*n* = 79)		*P* value	Perinatal mortality		*P* value
		Less than 7 (%)	7 or more (%)		Yes (%)	No (%)		Alive (%)	Mortality (%)	
Age (Years)	<30 years	12 (18.8)	52 (81.2)	0.544^#^	10 (16.7)	50 (83.3)	0.733^*∗*^	60 (93.8)	4 (6.2)	0.349^*∗*^
	≥30 years	5 (25.0)	15 (75.0)		4 (21.1)	15 (78.9)		17 (85.0)	3 (15.0)	
Antenatal care	Booked	3 (15.8)	16 (84.2)	0.544^*∗*^	2 (11.1)	16 (88.9)	0.504^*∗*^	17 (89.5)	2 (10.5)	0.654^*∗*^
	Unbooked	14 (21.5)	51 (78.5)		12 (19.7)	49 (80.3)		60 (92.3)	5 (7.7)	
Parity	Nullipara	8 (20.5)	31 (79.5)	0.953^#^	6 (17.1)	29 (82.9)	0.904^#^	35 (89.7)	4 (10.3)	0.699^*∗*^
	Multipara	9 (20.0)	36 (80.0)		8 (18.2)	36 (81.8)		42 (93.3)	3 (6.7)	
POG (weeks)	37–40	12 (17.4)	57 (82.6)	0.164^#^	11 (16.4)	56 (83.6)	0.437^*∗*^	65 (94.2)	4 (5.8)	0.104^*∗*^
	40^1/7^–42	5 (33.3)	10 (66.7)		3 (25.0)	9 (75.0)		12 (80.0)	3 (20.0)	
PROM	No	11 (18.6)	48 (81.4)	0.576^#^	10 (17.9)	46 (82.1)	1.000^*∗*^	54 (91.5)	5 (8.5)	1.000^*∗*^
	Yes	6 (24.0)	19 (76.0)		4 (17.39)	19 (82.6)		23 (92.0)	2 (8.0)	
Birthweight (gm)	<2500 gm	5 (26.3)	14 (73.7)	0.454^#^	3 (18.8)	13 (81.2)	1.000^*∗*^	15 (78.9)	4 (21.1)	0.043^*∗*^
	≥2500 gm	12 (18.5)	53 (81.5)		11 (17.5)	52 (82.5)		62 (95.4)	3 (4.6)	
Total		17 (20.2)	67 (79.8)		14 (17.7)	65 (82.3)		77 (91.7)	7 (83.3)	

^#^Chi-square test used; ^*∗*^Fischer exact test used.

## Data Availability

The hospital does not have a digital record of the data, and it was obtained by reviewing patient case files. The supporting data are available from the corresponding author upon request.

## Abbreviations

ACOG: American College of Obstetricians and Gynecologists
BPKIHS: B. P. Koirala Institute of Health Sciences
IUFD: Intrauterine fetal death
IUGR: Intrauterine growth restriction
LSCS: Lower segment cesarean section
LSOL: Latent stage of labor
MSV: Mauriceau–Smellie–Veit
NICU: Neonatal Intensive Care Unit
NND: Neonatal death
PROM: Prelabor rupture of membrane
RCOG: Royal College of Obstetricians and Gynecologists
TBT: Term Breech Trial.
